# Exploring learning communities’ actions and perceived impact on healthy weight approaches across Dutch municipalities

**DOI:** 10.1186/s12889-025-22072-0

**Published:** 2025-03-03

**Authors:** M. J.J. ter Bogt, K. E. Bevelander, G. R.M. Molleman, M. E.T.C. van den Muijsenbergh, G. A.J. Fransen

**Affiliations:** 1 Primary and Community Care, Radboud University Medical Centre, Nijmegen, The Netherlands; 2 AMPHI Academic Collaborative Centre, Nijmegen, The Netherlands

**Keywords:** Learning community, Collective action, Healthy weight, Community approach, Public health, Leverage point, Impact

## Abstract

**Introduction:**

Various Dutch municipalities apply healthy weight approaches (HWAs) to reduce overweight and obesity rates. Systems science theory suggests that HWA effectiveness may be increased by targeting leverage point themes (LPT) that drive change across HWAs. Actions relating to these LPTs can be applied by learning community (LC) members on different system levels varying from superficial to deep. However, there is a knowledge gap about how LCs can contribute to system changes and about LC members’ perception of their impact. Therefore, this study aims to gain insights into the perceived impact of these LCs on HWAs.

**Methods:**

This two-step study was performed in relation to two LCs and a steering committee executing actions over a 2.5-year period. At meetings, LCs created action lists (*n* = 8 and *n* = 2, respectively). We analyzed these action lists by identifying the corresponding LPTs, system levels, and perceived successful execution for each action, subsequently running frequencies and regression analyses. Additionally, we thematically analyzed qualitative evaluation questionnaires completed by LC members and the results of semi-structured interviews that we conducted with members.

**Results:**

Actions targeted all system levels and most LPTs at least once, including ten themes newly identified in this study. Actions that targeted the most superficial system level were most likely to occur. Knowledge exchange and combined forces LPTs were targeted the most. The communication strategies LPT was less likely to be executed successfully. Throughout later LC meetings, more actions were formulated regarding combined forces and central players LPTs, at the most superficial system level. Furthermore, members perceived impact on three main themes: ideas about the next steps toward solutions, more collaboration, and working more effectively.

**Discussion:**

Multidisciplinary LCs can contribute to creating new ideas and collaborations and to applying actions for a local HWA across most LPTs and system levels. These insights can be used during LC meetings to continuously adjust LCs and HWA actions when necessary. Future research is needed, for instance to gain further insights into new topics, the desired number of actions per LPT, and long-term LC impacts.

**Supplementary Information:**

The online version contains supplementary material available at 10.1186/s12889-025-22072-0.

## Background

Overweight and obesity are major health challenges of the 21st century, as worldwide 43% of the adults were overweight or obese in 2022 [1]. The Dutch government has set the goal of reducing the continuing rise in overweight and obesity rates in the Netherlands from 50 to 38% by 2040 [1–5]. Therefore, many municipalities collaborate with multiple local organizations from different sectors in applying healthy weight approaches (HWAs) [6, 7]. HWAs consist of policies, activities and interventions, and living environment facilities from an individual and an environmental perspective [6–13]. Yet, the effectiveness of such approaches remains limited [14–16], possibly because insufficient consideration is given to their complexity [17–19]. Therefore, improvements to HWA functioning are needed.

Interprofessional collaboration among diverse HWA stakeholders is essential to strengthen HWAs [12, 13]. Learning communities (LCs) are often established on purpose, and enhance interprofessional collaboration by providing informal spaces where members can learn from one another, strengthen collaboration, and align and adjust their actions [20–22]. LCs consist of a group of members that openly disclose their practices in an inclusive, continuous, reflective, and collective way with the aim of learning and growing [20, 23, 24]. For example, LC members can include a municipality’s policy advisors, municipal health service’s health brokers, care professionals (e.g., a general practitioner), practice professionals (e.g., a welfare worker), and citizens [25–27]. LCs are likely to produce a diversity of LC actions because of LCs’ multidisciplinarity. However, research is scarce on the perceived impact and actual impact of LCs. Previous research has shown that LC members’ perception of their LC’s impact is important for the continuation of LCs and for the successful execution of LC actions, as this perception strengthens members’ motivation to execute those actions [26, 27]. However, research is needed to understand how LCs strengthen HWAs and what members perceive as LC impact [26–28].

To take the complexity of obesity and HWAs into account, a systems perspective is required to monitor the LC impact [17, 18, 29–31]. A systems perspective embraces HWA complexity by acknowledging that all system elements are interrelated, unpredictable, hard to control, and adaptative to prevailing social norms, policies, and economic interests [17, 19]. A systems perspective also acknowledges the HWA as a system that consists of multiple levels [32, 33]. The Action Scales Model distinguishes four levels in a system [17]. The first layer– events, the most superficial level – includes stakeholders’ observable behaviors and outcomes within the system (e.g., general practitioners refer adults with obesity to combined lifestyle interventions). The next layer – structures – describes the organization of the system that causes these events to occur (e.g., general practitioners receive mandatory training about obesity complexity). The third layer describes the goals, which are the systems’ ambitions (e.g., all citizens have access to a general practitioner within one week). Finally, the deepest layer – beliefs – is comprised of stakeholders’ underlying norms, attitudes, and values about elements of the system (e.g., general practitioners reinforce across healthcare that obesity is the product of a complex system). As all four system levels are interconnected, changes in deeper layers may change more superficial layers as well [17].

In any of the system levels, general themes influence the functioning of the HWA system, including the HWA organizational structure, collaboration between professionals, and citizen participation [25]. Within these general themes, various, more specific, subthemes can be distinguished. For example, the HWA organizational structure consists of municipal processes; the diversity of themes, activities and tasks on which people work; networks; and communication strategies [25]. Within a subtheme, elements influence various other elements in the system. For example, the municipal processes theme consists of elements such as municipalities prioritize health, slow process, unclear HWA, financial resources, and perceived impact [25]. These elements describe topics to be targeted and are called leverage point themes (LPTs), as acting on them may cause changes throughout the entire HWA system [17, 25, 33]. LPTs thus indicate what changes (with regard to specific areas, systematic structures, and/or behaviors that contribute to the current problem) can be manipulated to promote resolution of the system; and overarch various smaller and more specific leverage points [17, 25, 34].

Actions in line with the relevant LPTs are needed to strengthen current HWAs and create systems change [17, 25]. Little is known about how LC actions relate to LPTs and system levels, and whether one type of action is more likely to be successfully executed than other types. Moreover, previous studies have not investigated whether different actions are formulated throughout the time course of LC meetings, yet differences possibly emanate from the complexity and adaptivity of HWAs and LCs. To understand the impact of LCs on strengthening HWAs, it is thus crucial to explore LC members’ perception of LC impact (perceived impact) as well as the extent to which LC actions contribute to the execution of LPTs across the system levels. Therefore, this study aims to provide insights into the perceived impact of LCs along two research objectives:

- To explore how LC members’ actions relate to the leverage point themes and system levels.
- To examine LC members’ perception of LC impact.

## Methods

### General study design

The study applied a mixed-methods design and consisted of two parts. Part 1 consisted of quantitative data regarding LC meeting action lists, and Part 2 consisted of qualitative interview data to examine LC members’ perceived impact. The Ethical Review Board of the Radboud University and Medical Center waived the need for a full review according to the Dutch Medical Research with Human Subjects Law (Wet Medisch-wetenschappelijk Onderzoek met mensen (WMO)) (registration number 2021–13172). We followed the ethical principles of the Declaration of Helsinki and GDPR regulations. All participants received an information letter and gave written informed consent.

### General study setting and study sample

#### Local LCs

Five Dutch municipalities received funding to start two LCs to strengthen current HWAs. A mainly fixed group of 10 to 20 HWA stakeholders per LC participated in a four-year project, including citizens, care professionals, practice professionals, municipality policy advisors, and municipal health service’s health brokers. The two LCs had one online and nine physical LC meetings each between October 2021 and June 2024. The first two LC meetings focused on members getting to know one another and the project start. From LC meeting three onwards, the observe-reflect-plan-act cycle was applied through reflexive monitoring [35, 36]. The LC methods and participants are described in more detail elsewhere [26].

#### Steering committee

The steering committee consisted of the municipalities’ health councilors, an LC member representative from each LC, advisors from national health parties, the LC facilitator/researcher (MB), and the project leader (GF). The majority of the municipalities’ health councilors were present at all four steering committee meetings organized between October 2021 and June 2024.

### Part 1: action lists

The study analyzed the action lists of eight LC meetings per LC and two steering committee meetings, as no action lists were created at the first two LC and steering group meetings.

#### Procedure

From LC and steering committee meeting 3 onwards, at the end of each LC meeting, all LC members present were invited to voluntarily formulate actions about what they were going to do with their insights obtained (see Additional file 1). Between a few minutes and a few days after the LC meeting, LC members filled in an evaluation questionnaire about the LC meeting [37], where LC members were asked to repeat their formulated action or formulate an additional action. There were only two cases where a specific member did not formulate an action during one specific LC meeting (98.8% of the cases formulated at least one action). These formulated actions were mapped on an action list (dynamic learning agenda) per meeting [36].

#### Data analyses and measures

**Fig. 1:**
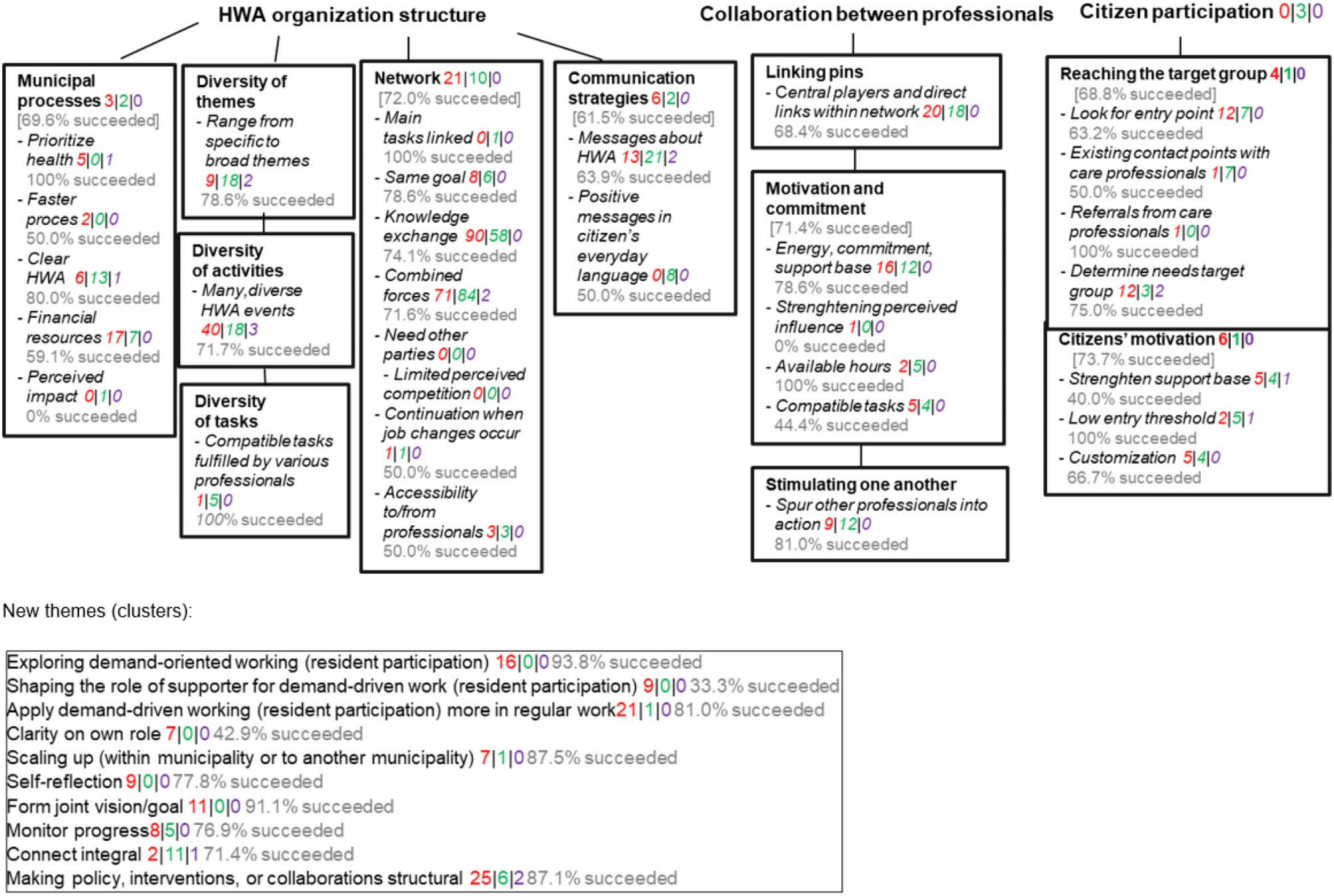
Overview of the (new) themes, underlying leverage point themes^a^, and the number of linked actions ^a^ Overview of the themes (bold), underlying LPTs* (in bullets), and the number of linked actions of respectively LC A (*n* = 340, red), LC B (*n* = 255, green), and steering group (*n* = 14, purple) meetings together. LPT definitions, operationalizations, and example actions are shown in Additional file 4 NB: Numbers indicated at the (sub)theme level correspond to actions that could not be attributed to underlying LPTs but clearly related to the (sub)theme NB: The percentages refer to the success rates of the goal attainment scale, indicating the percentage of the actions targeting that LPT that were deemed to be successful to a (much) greater extent than LC members had expected or as they had expected. The percentages between squared brackets indicate the sum of all LPTs across the subtheme. For example, the percentages between square brackets below the communication strategies subtheme includes the actions of both the messages about HWA and positive messages in citizens’ everyday language LPTs.

**Fig. 2:**
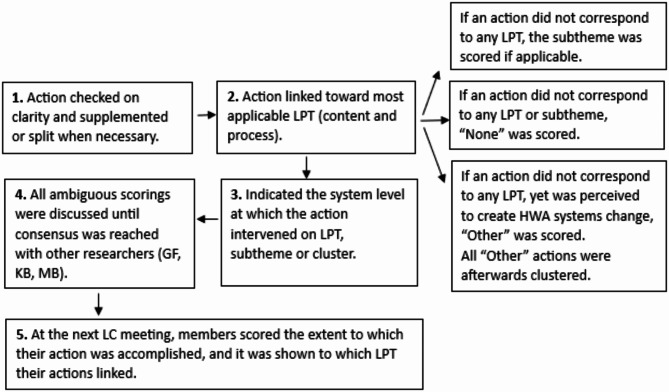
Diagram explaining the five steps to score the actions Note: LPT = leverage point theme

The main author (MB), who also facilitated all LCs, scored all LC actions on the LPTs [25] (displayed in Fig. 1) and system levels [17] in five steps, as explained in Fig. 2 (see Additional file 2 for more details). All actions were scored from a content and a process perspective. For example, the action, contact municipality for movement box subsidy, was linked to the financial resources LPT based on the content action (subsidy) and to the combined forces LPT based on the process action (contact municipality). The system level at which the action targeted the LPT, a subtheme, or a new cluster was determined for both the content and the process action. At the next LC meeting, the LC member who formulated the action indicated on an update form the extent to which the action was accomplished on the Goal Attainment Scale, which is a structured approach to indicate on a 5-point scale the extent to which the result of the action is achieved compared with expectations [38].^1^  

The following variables were scored per action (IBM SPSS Statistics 29): participant number, LC member function category (policy advisor, municipal health service's health broker, care professional, practice professional, citizen, health councilor), group (LC A, LC B, steering committee), meeting number (early – LC meetings 3, 4, 5, or steering group meeting 2022; middle – LC meetings 6, 7, 8, or steering group meeting 2023; late – LC meeting 9 or 10), goal attainment scale ((much) better result than expected (+ 2, + 1), expected result (0), (much) lower result than expected (-2, -1)) [39, 40], and the absence (0) or presence (1) of every LPT (formatted per (sub)theme) [25], the general category other, and system level [17].

Next, quantitative statistics were computed. First, frequencies of the actions’ system levels, LPTs, (sub)themes, and goal attainment scale were computed to understand the frequently formulated types of actions. Second, to understand what action characteristics were combined, crosstabs were computed between the actions’ (sub)themes/LPTs and system levels, (sub)themes/LPTs and (sub)themes/LPTs, (sub)themes/LPTs and goal attainment scale, system level and goal attainment scale, (sub)themes/LPTs and LC meeting number, and system level and LC meeting number. These crosstabs were used to identify the combinations of (sub)themes, LPTs, system levels, and LC meeting number that frequently occurred together, as they were to be used in further analyses (described below).

Third, to explore whether certain action types (system level, (sub)theme/LPT) were more likely to succeed, ordinal logistic regression analysis was computed, because it takes the ordinal nature of the goal attainment scale into account and estimates an odds ratio [41, 42]. LPTs with a higher number of occurrences may prove to be of importance in LCs. Therefore, the five LPTs with an n above 35 were also tested separately, whereas other LPTs were tested only on the (sub)theme level. Ordinal regression analyses were computed with goal attainment scale as dependent variable and each LPT/(sub)theme and system level as a separate predictor in another model, in light of the study’s explorative character.

Fourth, to test whether certain action types (system level, (sub)theme/LPT) were more likely to occur during early, middle, or late LC meetings, multiple simple binary regression models were computed where the (sub)themes/LPTs and system levels were separate dependent variables in another model, and the LC meeting number category (early, middle, late) was the predictor [43, 44]. When the model coefficients had a statistically significant value, polynomial, difference, or Helmert contrasts were computed to understand the patterns, depending on the observed patterns in the crosstabs. For all models, it was verified that none of the assumptions were violated; and odds ratios (OR) were calculated along their 95% confidence intervals.

### Part 2: perceived impact

We conducted semi-structured qualitative interviews and applied qualitative questionnaires at the end of every LC meeting.

#### Procedure

**Fig. 3:**
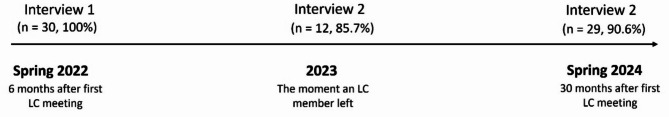
Timeline interview

All LC members were invited for two semi-structed interviews [26, 27], resulting in 71 LC members interviewed (93.4% response) (see Fig. 3). The first interview round took approximately 20 min and the second approximately 45 min. Most interviews were conducted by an independent interviewer (LT or IS), and in 2023 exit interviews (when LC members left the LC) were conducted by the main researcher (MB). A semi-structured interview guide was designed for all interviews and included questions regarding LC members’ perceived LC impact, as published elsewhere [26, 27]. Second, at the end of every LC meeting, LC members were invited to fill in an LC evaluation questionnaire that included open-ended questions regarding their perceived LC output [37].

#### Data analyses

Interviews were voice-recorded and transcribed verbatim. A thematic analysis using Atlas.ti version 9 software [45] was performed per LC on interview and qualitative questionnaire data in nine steps from the perspective of the question: *What is the perceived impact of the LC on the HWA?* (see Table 1 and Additional file 3 for more details).

**Table 1:** Nine steps, actions, aims, and results of the thematic analysis

Action	Aim	Result
**2022 interviews**		
1. Two coders (MB, EK) coded one transcript together and afterwards individually coded seven transcripts.	Identify relevant quotes and codes	First conceptual coding structure
2. One coder (EK) individually applied the coding structure to the remaining transcripts.^a^	Bottom-up coding of data	Finalized coding structure, 2022
***2023 data***		
3. The finalized 2022 coding structure was applied to 2023 interviews and open questions of the evaluation questionnaire for LC meetings 2 to 8 (MH). New codes appeared.^a^	Identify relevant quotes and codes	Conceptual coding structure, 2023
4. The codes were merged and clustered (MH, MB) and afterwards discussed until consensus was reached in the research team (MH, MB, KB, GF).	Develop the finalized coding structure for 2023	Finalized coding structure, 2023
***2024 data***		
5. The finalized 2023 coding structure was applied until barely no new codes were added, which took seven transcript among one LC and ten transcripts among the other LC (MB, LD).^a^	Identify relevant quotes and codes	Conceptual coding structure, 2024
6. The codes were merged and clustered (MB, LD) and afterwards discussed until consensus was reached in the research team (LD, MB, KB).	Develop the finalized coding structure for 2024	Finalized coding structure, 2024
7. Two coders (MB, LD) applied the clustered coding structure to the remaining transcripts, and three coders (AR, TH, MB) to the evaluation questionnaire for LC meetings 9 to 11.^a^	Bottom-up coding of data	Fully coded data
***All data***		
8. The identified clusters were discussed, ordered, and categorized in main themes and subthemes (MB, EK, MH, LD, AR, TH).	Identify possible subthemes within each main theme	A taxonomy of main themes and possible subcategories
9. The groups of codes, themes, and subthemes were discussed by the research team until consensus was reached (MB, EK, MH, LD, AR, TH, KB, GF).	Identify definite subthemes and their mutual relations within each main theme	Main themes and their subthemes as presented in the results section

a Throughout the entire process, new codes and doubts were discussed with the second coder (MB). Further, to ensure validity among all coders, various random checks were performed (MB) when the coders indicated that all transcripts were adequately coded

## Results

### Part 1: action lists

**Fig. 4:**
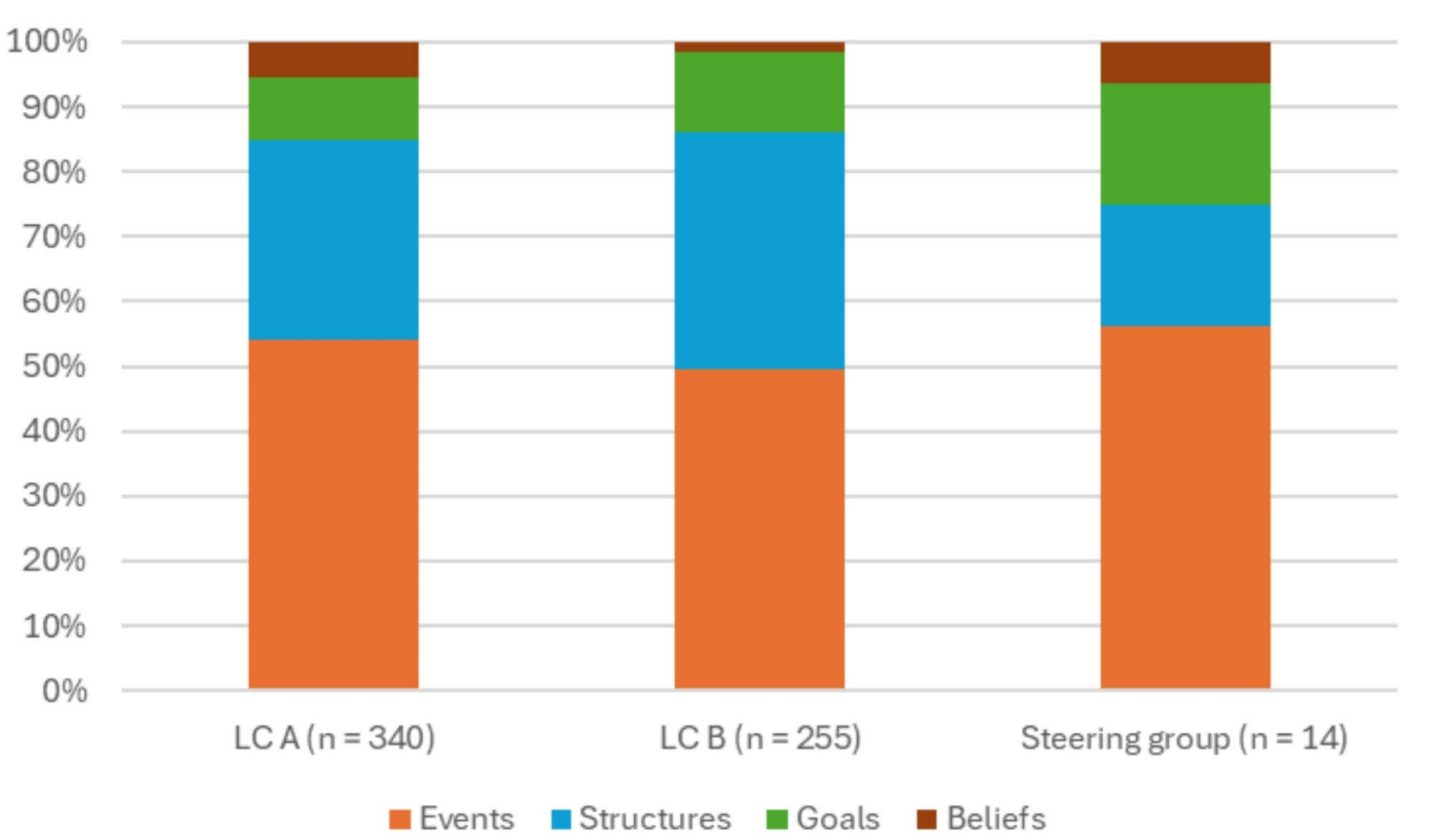
Overview of the targeted system levels per group (all meetings totaled)

In total, 609 actions were formulated. LC members formulated on average 4.0 (standard deviation 2.4) actions per LC member per meeting, and steering committee members formulated on average 1.4 (standard deviation 0.7) actions per member per steering committee meeting. Below we describe the extent to which these actions targeted the relevant topics (i.e., LPTs; Figs. 1 and 4) and the corresponding level on which they targeted them (i.e., system levels; Fig. 4).

#### Actions targeting leverage point themes

Figure 1 indicates per group (LC A, LC B, steering group) how many formulated actions targeted the LPTs across all meetings (see Additional file 4 for example actions per LPT).

##### HWA organizational structure

Over all groups (LC A, LC B, steering group), most actions targeted the network subtheme (*n* = 346 of 609 actions). Some actions targeted the municipal processes (*n* = 58), diversity of activities (*n* = 61), diversity of themes (*n* = 29), or communication strategies (*n* = 52) subthemes. The diversity of tasks subtheme (*n* = 6) was barely targeted. More specifically, the knowledge exchange (*n* = 148) and combined forces (*n* = 152) LPTs were targeted the most by both LCs. Further, all groups regularly targeted the financial resources (*n* = 24), messages about the healthy weight approach (*n* = 36), many diverse HWA events (*n* = 61), and range from specific to broad themes (*n* = 29) LPTs. However, LPTs such as compatible tasks fulfilled by various professionals (*n* = 6), faster process (*n* = 2), and limited perceived competition (*n* = 0) were barely targeted.

##### Collaboration between professionals

Some actions targeted the linking pins (*n* = 38), motivation and commitment (*n* = 45), and stimulating one another (*n* = 21) subthemes. More specifically, both LCs regularly targeted the central players and direct links within the network (*n* = 38), spur other professionals into action (*n* = 19), and energy commitment and support base (*n* = 28) LPTs. However, the other LPTs regarding motivation and commitment were barely targeted.

##### Citizen participation

Several actions targeted the reaching the target group (*n* = 49) and citizens’ motivation (*n* = 34) subthemes. More specifically, all groups regularly targeted the look for entry point (*n* = 19) and determine needs target group (*n* = 17) LPTs. However, LPTs such as referrals from care professionals (*n* = 1) and customization (*n* = 9) were barely targeted. Further, the existing contact moments with care professionals (*n* = 8) LPT was targeted almost solely by LC B.

#### Actions targeting new themes

The actions in the category other did not correspond to any LPT but were perceived to create HWA systems change encompassed 10 clusters (Fig. 1). Three clusters related mainly to citizen participation and were targeted mainly by LC A; these were exploring demand-oriented working (citizen participation), shaping the role of supporter for demand-driven work (citizen participation), and apply demand-driven working (citizen participation) more in regular work. Other clusters applied mainly in LC A were clarity on own role, scaling up (within municipality or to another municipality), self-reflection, and form joint vision/goal. Moreover, the clusters targeted in both LCs were monitor progress, connect integral, and making policy, interventions, or collaborations structural, of which the last two were also targeted by steering committee members. Altogether, LC A targeted these new clusters relatively often, whereas LC B barely did.

#### Actions targeting system levels

Figure 4 indicates that, in all groups, the majority of actions targeted system level events (*n* = 401 of 609 actions). The structures level was also targeted repeatedly (*n* = 253), whereas the system-level goals (*n* = 84) and beliefs (*n* = 30) were barely targeted. Actions regarding many LPTs and clusters roughly represented the system levels in a comparable pattern as shown in Fig. 4, such as many, diverse HWA activities and knowledge exchange. Still, some seemed to have slightly different patterns. For instance, actions regarding the central players and direct links within the network LPT targeted events (29%), structures (46%), goals (18%), and beliefs (6%). Most actions across all system levels were executed as expected or (much) more than expected: 75.3% for actions targeting events, 68.6% for structures, 70.7% for goals, and 80.0% for beliefs.

#### Successfully executed actions

Across almost all LPTs/subthemes, new themes, and system levels, most actions were perceived as successful (Fig. 1). Overall, 26.1% of the actions were perceived as much more or more successful than LC members had expected, 46.2% as expected, and 27.8% much less or less than expected (*n* = 598) (response 98.2%). Multiple simple ordinal regression analyses indicated two statistically significant models, suggesting that actions that targeted system-level events seemed more likely – whereas actions that targeted communication strategies subtheme seemed less likely – to be executed successfully compared with actions that did not (Table 2; Fig. 1). For example, actions that did not target system-level events were associated with a 0.406 decreased likelihood of the action being executed successfully compared with actions that did target system-level events (Table 2).

**Table 2:** Multiple simple ordinal regression analyses with reported successful action as outcome

Variable	Regression coefficient	Standard error	Wald	OR	95% CI for OR
Communication strategies = 0 (subtheme)	0.547	0.273	4.019	1.728***	1.012–2.949
Events = 0 (level)	-0.520	0.162	10.271	0.594**	0.432–0.817

OR = Odds ratio; CI = Confidence interval

*p < 0.001; **p < 0.01; ***p < 0.05

Both models had a model of fit (*p*-value < 0.05), goodness of fit (*p*-value > 0.05), and test of parallel lines (*p*-value > 0.05)

#### Different actions throughout LC meetings

Some types of actions seemed more or less likely to be formulated specifically at early, middle, or late LC meetings, compared with other LC meeting numbers (Table 3). Multiple simple binary regression analyses suggested that LC meeting number seemed to account for a significant amount of variance for several outcome variables (Table 4). Contrast analyses (Table 3) illustrate, for example, that statistically significantly more LC actions targeted events during middle (74.6%) and late (70.6%), compared with early, LC meetings (43.2%). This means that actions formulated in the middle or late LC meetings had a 1.793 increased likelihood of these actions targeting system-level events compared with the odds for the early LC meetings (Tables 3 and 4).

**Table 3:** Results of contrast analyses, including the number (percentage) of actions formulated per LC meeting category

Variable	Early	Middle	Late
Collaboration between professionals (theme)	**14 (9.6%)**	55 (19.4%)	34 (18.9%)
Network (subtheme)^a^	**73 (50.0%)**	**157 (55.5%)**	**116 (64.4%)**
Linking pins (subtheme)	4 (2.7%)	11 (3.9%)	**23 (12.8%)**
Combined forces (leverage point theme)^a^	**21 (14.4%)**	**78 (27.6%)**	**58 (32.2%)**
Central players and direct links within the network (leverage point theme)	4 (2.7%)	11 (3.9%)	**23 (12.8%)**
Events (level)	**63 (43.2%)**	211 (74.6%)	127 (70.6%)
Goals (level)^a^	**29 (19.9%)**	**38 (13.4%)**	**17 (9.4%)**

The percentage is the number of actions formulated in that theme, subtheme, or system level compared with all actions formulated at early, middle, or late LC meetings. Bold numbers represent a statistically significant higher or lower likelihood of formulating an action on a variable in an LC meeting number category (early, middle, late) compared with the other LC meeting number categories

^a^ For three variables, contrast analyses suggested an increasing or decreasing trend across all three meeting number categories

**Table 4:** Results of statistically significant simple binary logistic regression analyses with LC meeting category as predictor

Variable	Regression coefficient	Standard error	Wald X^2^	OR	95% CI for OR
Collaboration between professionals (theme)	0.318	0.151	4.418	1.374***	1.022–1.848
Network (subtheme)	0.300	0.113	7.001	1.349**	1.081–1.685
Linking pins (subtheme)	0.995	0.268	13.779	2.705*	1.600–4.575
Combined forces (leverage point theme)	0.467	0.131	12.620	1.595*	1.233–2.064
Central players and direct links within the network (leverage point theme)	0.995	0.268	13.779	2.705*	1.600–4.575
Events (level)	0.584	0.122	23.083	1.793*	1.413–2.276
Goals (level)	-0.436	0.164	7.104	0.646**	0.469–0.891

OR = Odds ratio; CI = Confidence interval

* *p* < 0.001; ** *p* < 0.01; ***p < 0.05

### Part 2: LC members’ perceived impact

**Fig. 5:**
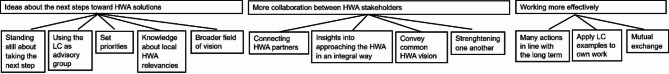
Overview of LC members’ perceived LC impact themes and subthemes

Perceptions about LC impact varied across LC members, as LC members stated that their experience ranged from a lot to only small LC impacts; and the types of impact mentioned were not recognized by all members. Overall, LC members perceived LC impact regarding three main themes: ideas about the next steps toward solutions, more collaboration, and working more effectively, which all consisted of subthemes (as illustrated in Fig. 5). These themes corresponded to subsequent impact levels that strengthened one another. For example, ideas about the next steps contributed to more collaboration; and both ideas and collaboration contributed to working more effectively.

#### Ideas about the next steps toward solutions

The LC provided members with ideas about the next steps to strengthen their HWA work, and this was perceived as an LC result. To get to these insights, four different elements were mentioned. First, LC members used the LC as a moment to stand still and think about taking the next step together with other members; this moment of reflection was valued, as it mitigated the day-to-day business. Besides, the LC was used as an advisory group where members took the opportunity to introduce their own messages or connect with people who could help with their own action. Second, throughout this process, the LC helped members to prioritize prevention and realize that small actions are important. This helped members to set priorities:

*I think it helps me to see perhaps confirmation of certain ideas that may be going on somewhere in the background*,* like okay*,* yes*,* that is indeed useful to do. And then to prioritize that. So in that way it helps me to prioritize.… In addition*,* I can imagine that it can be a bit of a sticky factor for everyone to keep the theme on their minds all the time.* (Policy advisors)

Third, throughout this process, LC members gained knowledge about local HWA relevancies relating to knowledge about HWA content (i.e., knowledge about what HWA stakeholders do), knowledge about HWA success factors (i.e., involving the target group requires personal contact and a question-centered way of working), knowledge about HWA problems (i.e., the target group, young adults, is barely incorporated in the HWA), and awareness of the HWA’s complexity. According to members, this knowledge originated from hearing research results and perspectives of members who were outside their direct work environment. Lastly, LC members mentioned that the LC contributed to a broader field of vision, for example by seeing the bigger HWA picture and understanding other HWA stakeholders better (i.e., increasingly speaking the same language and empathizing with one another):

*That you now actually look a little more broadly than your own target group*,* but just really take a municipal look*,* okay*,* what can we all offer somewhere?* (Practice professionals)

#### More collaboration between HWA stakeholders

Some ideas about the next steps related to collaborating with HWA stakeholders. The LC resulted in more collaboration between HWA stakeholders. Four aspects were mentioned as achieving this collaboration. First, members’ connection with HWA stakeholders ultimately contributed to collaboration:

*So*,* you get closer to one another and that connection is an important point for me at least. What you see when you look here is that people often work next to one another and past one another and that in such a learning community you do get more connection with one another.* (Citizen)

Through the LCs, members learned how to organize HWAs together (i.e., knowledge about the municipality’s policy choices) and created a broader network (i.e., contact with local public, working more with the municipality), because the LC brought members into contact with people whom they otherwise would not have met. Moreover, members shared the LC ideas with their own network, thereby further connecting members with HWA stakeholders.

Second, the LC enabled members to appreciate a more integral perspective on health and the HWA (i.e., looking more broadly at health and their target group). Consequently, members realized that they needed other stakeholders and therefore connected HWA elements during the LC, thereby further enabling collaboration. Third, members experienced a common HWA vision and gained trust in strengthening the HWA. Lastly, at LC meetings, members experienced more social support among themselves, energy to work on the HWA, and usage of one another’s expertise (e.g., knowledge about how others work on specific HWA themes), again further strengthening collaboration:

*What I like is what I noticed last time: you pose a problem in the group*,* there are network partners around you who say oh hey*,* that’s what I’m here for*,* but I can help with that.* (Practice professional)

*It gives me energy. … You can then use the energy in the learning community directly for what will be rolled out in the municipality.* (Health broker)

#### Working more effectively

Some collaborations related to working effectively. LC members worked more effectively (i.e., more efficient regular meetings) by applying three types of actions, where actions include for instance LC ideas, projects, or working methods. First, in line with increased collaboration, working more effectively was enabled through actions about mutual exchange. LC members had conversations with members outside the LC meeting, and LC members knew what the other members could offer. Second, various LC members applied a long-term view to envisioned actions (i.e., a prevention team was initiated in the municipality). Third, members partly organized their work more in line with citizens’ needs, thereby strengthening the position of citizen participation, and members considered this step crucial for effective work. The diversity of HWA stakeholders implied variety in the way in which members applied actions in their regular work. Examples included identifying citizens’ needs (practice professional), creating policy plans in which citizens could be involved (policy advisor), and tailoring advice to patients (care professional). Thus, members mentioned successful LC projects (e.g., published booklet, development of communication, initiatives copied from another municipality), and others indicated that they had adapted their behavior during regular work, such as consultations (i.e., referring more to neighborhood initiatives):

*Well*,* for example*,* in our Healthy and Active Living Agreement we have included something for that*,* that in the process we are now going through around the Healthy and Active Living Agreement we will look at how we can involve citizens and target groups adequately*. (Policy advisors)

*Um well*,* in the last years*,* I have been looking a lot more*,* not what can I do for the patient*,* but what does a patient need to help him/herself. I think that is much more important and has become for me*,* and also not provide some standard exercises. But much more looking at what someone is doing and much more adapting exercising accordingly*. (Care professional)

## Discussion

The current study adopted two approaches to explore the perceived impact of learning communities (LCs) on healthy weight approaches (HWAs). Approach 1 investigated how LC actions relate to the themes that drive change across HWAs (i.e., LPTs) and their system levels. Approach 2 consisted of qualitative interviews with LC members on their perceived LC impact. Given the richness of our study, we discuss the following five findings that address our research objectives. The results of both approaches overlapped, meaning that the actual actions formulated by members throughout LC meetings corresponded to the LC impacts on HWAs experienced by the members. Moreover, LC members’ actions (objective 1) related to LPTs and ten new themes. Further, actions that related to the most superficial system level were more often formulated and more likely to be executed successfully compared with actions that did not. Moreover, some types of actions were more likely to be formulated during later, compared with earlier, LC meetings. All in all, LC members perceived (objective 2) that multidisciplinary LCs contribute to acquiring ideas, creating collaborations, and applying actions for a local HWA.

First, members’ LC actions matched with their perceived LC impact on the HWA. Our study demonstrates that the knowledge exchange, combined forces, and central players LPTs were targeted the most through actions; this corresponded to one of the main LC impacts mentioned: collaboration. Other types of LC impacts mentioned related to ideas about the next steps and working more effectively, also corresponding to the actions. Previous research across communities of practice identified comparable LC impacts on public health, such as continuous learning, networking to keep up-to-date in the field, and increased productivity and quality of work [46, 47]. Further, most previously reported success measures for community-based dynamic public health efforts were also identified in our LC impact on HWAs, such as individual thinking (e.g., broader field of vision), collaborative experience (e.g., strengthening one another), ownership, trust, and relationships (e.g., convey common HWA vision), and prevention practice (e.g., apply LC examples in own work) [48]. Some success measures were not identified among our LCs, such as health outcomes and unintended consequences [48]. As these success measures are often recognized as long-term outcomes [18, 48], it is possibly necessary to monitor LCs for a longer period to identify these. Future research is needed to gain insights into longer-term LC impacts on HWAs and to understand the pathways toward achieving them, including how LC impacts are caused and interrelated.

Second, our findings show that, even though actions targeted most LPTs, some LPTs were not – or only limitedly – targeted, such as continuation when job changes occur (network) and positive messages in citizens’ everyday language (communication strategy). There are several potential explanations for this. First, the LC content (conversations, reflections, and/or partners present) may have related insufficiently to these LPTs, making it unlikely that actions about these LPTs would be formulated. Second, multiple LC members mentioned that these LPTs were already targeted in their regular work outside the LC and therefore not in the LC. For example, one municipality focused on its communication strategy messages in citizens’ language in collaboration with an external communication agency. Third, prerequisites for these LPTs might be insufficiently met, such as members not feeling motivated or responsible, no supportive backup, municipality’s policy operating inadequately, and limited sphere of influence [25–27, 49, 50]. Actions that targeted the communication strategies subtheme seemed less likely to be executed successfully. Perhaps actions regarding communication strategy messages in citizens’ language were targeted less because they were perceived as being more difficult to apply. Lastly, it is unlikely that the same number of actions are needed to implement every LPT. Therefore, future research is needed to better understand what the number of actions applied per LPT might indicate.

Third, this study identified ten new themes targeted through actions in addition to the LPTs investigated two years earlier among the same HWA stakeholders [25]. These new themes were perceived as important for members to create HWA systems change. These new themes may reflect learning among members, as learning improves HWA system awareness. Possibly, these new themes reflect further developed HWAs, meaning that the themes were not yet relevant during the earlier interviews. Future research is needed to understand whether and to what extent these new themes indicate growth, what LPTs correspond to these new themes, and how these new themes relate to the previously identified LPTs.

Fourth, LC actions targeted mainly the superficial system level. Actions that targeted superficial system-level events were significantly more likely to be executed successfully compared with actions that did not. This is partly in line with the Action Scales Model [17], which states that actions targeting superficial system levels are easier to execute but require more actions to create systems change compared with actions targeting deeper system levels [17]. Our research adds that actions targeting the events level seem more likely to yield results in practice compared with actions that do not. This is important, as previous research indicates that members need to experience action results to remain motivated to execute actions and participate in LCs [26]. This may explain why most LC actions targeted the superficial system level and why targeting the events level in addition to deeper system levels may be relevant to create systems change. Given the limited number of steering group actions and actions that targeted the deeper system levels, future research with more LCs is needed to further understand these types of actions. Moreover, more insights into interdependencies of action list characteristics (e.g., job functions) are desired to further understand action patterns.

Lastly, we found that, throughout later LC meetings, more actions were formulated regarding the combined forces and central players LPTs and the events level compared with earlier meetings. This may indicate that LC members gained more ownership throughout later LC meetings, resulting in more space for spontaneous networking with members during meetings. Earlier research indicates that members prefer networking in LCs [26] and that networking supports knowledge exchange and motivation to collaborate [51, 52]. This may explain why more actions were formulated toward these leverage points. To enable members to *also* execute actions that target other LPTs, members probably need to be continuously stimulated to reflect on what actions are likely to lead to meaningful change [17, 18]. Therefore, members need to understand the importance of systems thinking and have the skills to apply it in practice [53, 54]. To achieve this, LCs need to be extensively facilitated from a systems perspective, possibly including LC meeting aspects that initially seem to contradict members’ preferences as they are learning to apply systems thinking. Future research is thus needed about how to improve the application of systems thinking in multidisciplinary LCs. Still, members perceived that some systems change took place in practice. For instance, some policy change took place through adding citizen involvement to the municipality’s Healthy and Active Living Agreement.

Altogether, HWA stakeholders are recommended to create, and participate in LCs or comparable working groups to get ideas, collaborations, and work more effectively by executing actions that address the LPTs and new topics. When doing so, LC facilitators and members are recommended to determine and subsequently focus on LPTs and system levels that have been limitedly applied in regular work practices so far.

### Strengths and limitations

The study’s strengths include high participation rates, ensuring that all relevant actions were captured. Further, our study had an adequate ecological validity, meaning that the study setup matched with LC members’ real work context. For example, within the LCs, no money was available to execute actions, meaning that all actions were applied through regular work; this increases the generalizability of our findings.

One of the study’s limitations is that LC actions did not always conform with the SMART formula (specific, measurable, acceptable, realistic, and time-bound); action list analyses were therefore limited. Further, some actions were repeatedly partly executed and therefore reformulated at a subsequent meeting, whereas others were executed straight away. Therefore, the number of times an LPT was targeted may be overrepresented, and the result must be interpreted carefully. Moreover, the extent to which some LC actions would possibly have been implemented without the LC is also unclear. Still, LPTs that were frequently targeted through actions were in line with members’ perceived LC impact; this increases the likelihood that the LC contributed to the implemented actions.

## Conclusion

The current study gained unique insights into short-term LC outcomes, leading to possible HWA systems change. Our study indicates that multidisciplinary LCs can contribute to acquiring ideas and collaborations and applying actions for a local HWA, through targeting LPTs and new themes on all – but mostly the most superficial – system levels. LCs are recommended to monitor and use these short-term outcomes during LC meetings to adjust LCs and HWA actions, enabling continuous adaptivity, which is essential in complex systems like HWAs. Researchers are recommended to apply and further develop these methods to understand LC outcomes toward HWA systems change.

## Electronic supplementary material

Below is the link to the electronic supplementary material.

Supplementary Material 1

Supplementary Material 2

Supplementary Material 3

Supplementary Material 4

## Data Availability

The data that support the findings of this study are available from Radboudumc but restrictions apply to the availability of these data, which were used under license for the current study and so are not publicly available. Data are, however, available from the authors upon reasonable request and with permission of Radboudumc. Therefore, data may be requested by emailing the quality team of our department of primary care (kwaliteitsteam.elg@radboudumc.nl).

## Footnotes

1 At LC meeting 3, however, LC members did not score their own action. Instead, all municipal health service's health brokers who were present scored all LC members’ actions individually; consequently, the scores were averaged and afterwards rounded to get one score per action.
