# Supplementary material for: Exploring learning communities’ actions and perceived impact on healthy weight approaches across Dutch municipalities
Source: BMC Public Health. 2025 Mar 3;25:839. doi: 10.1186/s12889-025-22072-0 (PMC11874444; doi:10.1186/s12889-025-22072-0)
Supplement: Supplementary file 1 — Supplementary Material 1 [file 12889_2025_22072_MOESM1_ESM.docx]

**Additional file 1: Action formulation form for completion at the end of an LC meeting**

Name:

What are you going to do (differently) before the next learning community meeting?

|  |
| --- |

Why are you going to do this?

|  |
| --- |

How are you going to do this? What or who do you need for this?

|  |
| --- |

How do you involve your colleagues/constituency in this?

|  |
| --- |
