# Supplementary material for: Exploring learning communities’ actions and perceived impact on healthy weight approaches across Dutch municipalities
Source: BMC Public Health. 2025 Mar 3;25:839. doi: 10.1186/s12889-025-22072-0 (PMC11874444; doi:10.1186/s12889-025-22072-0)
Supplement: Supplementary file 2 — Supplementary Material 2 [file 12889_2025_22072_MOESM2_ESM.docx]

**Additional file 2: Data analyses action lists**

The main author (MB) who also facilitated all LCs scored all LC actions in five steps. First, all actions were checked on clarity, and supplemented where necessary based on LC meeting minutes and voice records to ensure adequate interpretation. If a formulated action consisted of multiple actions, these were split up into separate actions. Second, the action was, when possible, linked towards the leverage point theme that was the most applicable for the content action, and when necessary another leverage point theme for the process action (Figure 1; Additional file 4) (ter Bogt et al., 2023). For example, the action “Contact municipality for movement box subsidy” was linked to leverage point theme “Financial resources” based on the content action (“subsidy”), and the leverage point theme “Combined forces” based on the process action (“contact municipality”). If an action did not correspond to any of the leverage point themes, but did correspond to the broader subtheme that subsisted of specific leverage point themes, that subtheme was scored instead. For example, the action “Meet new [employee at organization] (…) Antenna on for possibility” was not linked towards any of the leverage point themes, but was linked towards the subtheme “Network”. If an action did not correspond to any of the leverage point themes nor subtheme, yet seemed important to create systems change within HWAs according to LC members, it was labeled as “Other”. These “Other” actions were clustered across both LCs, and scored likewise. Third, it was indicated on what system level (events, structures, goals or beliefs) the action targeted the leverage point theme or group (Nobles et al., 2021). Fourth, to increase validity, all ambiguous scorings were discussed until consensus with two other researchers (MB, GF, KB) within two weeks after the LC meeting took place. Fifth, during the next LC meeting, the LC member who formulated the action indicated on an update form to what extent the action was accomplished on the Goal Attainment Scale, which is a structured approach to indicate on a 5-point scale to what extend the result of the action is achieved compared to their expectations (Bovend’Eerdt et al., 2009).^[[1]](#footnote-1)^ Lastly, to increase validity, LC members were shown to what leverage point theme their action linked, and a LC wide overview was presented regarding how often a leverage point theme was scored among the actions of the previous LC meeting action list.

1. Only among LC meeting 3 LC members did not score their own actions, instead all present municipal health service’ health brokers scored all LC members’ actions individually, subsequently the scores were averaged and afterwards rounded to get one score per action. [↑](#footnote-ref-1)
