# Supplementary material for: Exploring learning communities’ actions and perceived impact on healthy weight approaches across Dutch municipalities
Source: BMC Public Health. 2025 Mar 3;25:839. doi: 10.1186/s12889-025-22072-0 (PMC11874444; doi:10.1186/s12889-025-22072-0)
Supplement: Supplementary file 3 — Supplementary Material 3 [file 12889_2025_22072_MOESM3_ESM.docx]

**Additional file 3: data analyses perceived impact**

Interviews were voice-recorded and transcribed ad verbatim. A thematic analysis was applied among interview, and qualitative questionnaire data per LC from the perspective of the question: *What is the perceived impact of the LC on the HWA?* by using Atlas.ti version 9 software (Braun et al., 2006). To develop a coding structure, two coders coded one transcript of spring 2022 together and afterwards individually coded seven transcripts, which were discussed and merged afterwards until consensus with a third researcher (MB, KB, EK). Next, one coder (EK) individually applied the coding structure to the remaining transcripts of spring 2022 (EK), of which new codes and doubts were discussed with the second coder (MB, EK). The final coding structure of the transcripts of spring 2022 was applied to the exit transcripts of 2023, and open questions of the evaluation questionnaire of LC meeting 2 until 8 (MH). If new codes emerged, these were discussed with the second coder (MB), which resulted in a final coding structure of the 2023 exit interviews (MB, MH). Next, the codes were merged and clustered (MH, MB) and afterwards discussed until consensus with the research team (MH, MB, KB, GF), as described below. These final clusters were applied to the transcripts of 2024 (MB, LD), and open questions of the evaluation questionnaire of LC meeting 9, 10 and 11 (AR, TH, MB). If new open codes emerged, these were discussed with the second coder (MB), which resulted in a final coding structure of the 2024 interviews (MB, LD, AR, TH, KB). Further, among every data time point (spring 2022, exit, spring 2024) random checks (MB) where performed when the coders indicated that all transcripts were adequately coded to ensure validity among all coders.

Next, the coded data of spring 2022 were grouped into subthemes (MB, EK, KB). For example, codes such as “along the same lines”, “action convey vision”, “collectivity” were grouped into the subtheme “convey collective vision HWA”. Next, the identified subthemes were discussed, ordered and categorized in main themes per LC group (MB, EK, KB). Afterwards, these (sub)themes were updated among new codes from 2023 exit interviews and corresponding evaluation questionnaires (MB, MH, KB), and later again among new codes from spring 2024 interviews and evaluation questionnaires (MB, LD, AR, TH, KB). Subsequently, already existing (sub)themes were expanded and/or renamed, and new (sub)themes appeared. For example, codes such as “priorities”, “clear possible solutions”, “knowing next steps”, “insights tools for initiatives in the social sphere” were grouped into the subtheme “insights into solutions/next steps”, while these did not exist in 2022 data.
