# Supplementary material for: Exploring learning communities’ actions and perceived impact on healthy weight approaches across Dutch municipalities
Source: BMC Public Health. 2025 Mar 3;25:839. doi: 10.1186/s12889-025-22072-0 (PMC11874444; doi:10.1186/s12889-025-22072-0)
Supplement: Supplementary file 4 — Supplementary Material 4 [file 12889_2025_22072_MOESM4_ESM.docx]

**Additional file 4: Leverage point theme (LPT) and system level definitions, operationalizations, and examples**

| **LPT (ter Bogt et al., 2023)^a^** | **Definition (ter Bogt et al., 2023)^a^** | **Example action** |
| --- | --- | --- |
| 1.1 Prioritize health | Municipalities prioritize health (e.g., policy documents about healthy weight). | Health urgency spot on the horizon. |
| 1.2 Faster process | Municipal processes are perceived as supportive by being faster. | [Approach again. Next step [project name]. Working with new material and gaining experience for [project name]*:* follow up with the municipal project team and policy civil servant. Awaiting approval for use of public space. Continue with [project name] as soon as the municipality approval arrives. |
| 1.3 Clear HWA | HWAs are perceived as clear (e.g., municipality has adequately aligned topics and visions). | Determine direction regarding developments (Healthy and Active Living Agreement (GALA), and associated specific allowance (SPUK)) |
| 1.4 Financial resources | Sufficient financial resources are available (i.e., sufficient working hours and sustainability of events). | Consultation with municipalities on the embedding of policy and finance for the purpose of a structural approach: trying to sharpen the goals, resources, and profit for policymakers so that expenditures now become profit in the future. |
| 1.5 Perceived impact | Professionals perceive that they have impact with their HWA contribution (i.e., because of structural funding and expressing the HWA’s impact in terms of citizens’ health gains). | Thinking about how the benefits from preventive health programs can be better captured. Experience stories, exercise ambassadors, and bundling the number of members. Map how many extra sport association members the campaign [name] has delivered and people’s stories to see what behavior change has occurred as a result of the exercise campaign. |
| 2.1 Range from specific to broad themes | HWA themes range from specific (e.g., more exercise and healthier eating) to broad (e.g., broad development among children, meaningfulness in life). | Connecting the healthy weight theme with other themes. |
| 3.1 Many diverse HWA events | Many and diverse initiatives and activities are organized, thereby facilitating the integral aspects of the HWA (i.e., activities, working groups, daily work activities, other). | Investing in young people’s mental health. Establishing contacts with [function names] to (further) develop the 12+ offers. |
| 4.1 Compatible tasks fulfilled by various professionals | HWA execution is diverse and compatible because many different organizational tasks in the HWA are fulfilled by different professionals because their regular tasks differ. | Support [project name]. |
| 5.1 Main tasks linked | Contact between professionals by having a main task that links directly to another stakeholder’s task. | Continuing this way [More actively taking photos with colleagues to share. Working with MailChimp for more monthly overviews. Discuss with colleagues what has been done] and continue to collect photos and activities with all colleagues. |
| 5.2 Same goal | Contact between professionals by working on the same goal. | Initiate consultation with [names] about the goal and vision of a healthy weight approach. |
| 5.3 Knowledge exchange | Contact between professionals by exchanging knowledge facilitates. | Distribute knowledge and skills where necessary. |
| 5.4 Combined forces | Contact between professionals by combining forces to complement one another. | Intensify contact with primary care professionals regarding prevention/healthy lifestyle, together with a health broker. Where is support needed and what role can respond to better collaboration with primary care professionals? |
| 5.5 Need other parties | Contact between professionals by feeling that other parties are needed. |  |
| 5.6 Limited perceived competition | Contact between professionals by experiencing limited competition or believing that a target group can be targeted by multiple professionals. |  |
| 5.7 Continuation when job changes occur | Contact between professionals by adequate continuation when job changes occur. | Meet the new (temporary) social team coordinator to further discuss [build on the go/no go prevention team] in combination with further development of social core team. |
| 5.8 Accessibility to/from professionals | Contact between professionals by accessibility to/from professionals (e.g., responding to questions, feeling that the professional has time, structurally having contact with one another). | Make plan of campaign together with [name] to get in contact with primary care professionals in our municipality [strengthen the connection between 0^th^ and 1^st^ line]. |
| 6.1 Messages about HWA | Communicating messages about HWA activities to citizens and professionals via various communication channels, for example local television, newspapers, the internet, or care professionals, with the aim of creating familiarity about activities in municipalities. | Develop and implement communication plan. |
| 6.2 Positive messages in citizens’ language | Communication strategies include positive messages in citizens’ everyday language, not formulated in terms of weight and not patronizing, and spread at places frequented by citizens. | Consultation with [names] regarding possibility to flesh out the website (after communication advice). |
| 7.1 Central players and direct links within network | Linking pins, also called central players, facilitate collaboration between professionals by having short communication lines with other stakeholders. | Perhaps take the role of connector even more strongly. |
| 8.1 Energy, commitment, support base | Being enthusiastic about working on specific HWA tasks and experiencing a lot of energy, commitment, and support among other professionals. | Lobbying in my immediate environment. |
| 8.2 Strengthening perceived influence | Strengthening the perceived influence on the HWA by acting within one’s sphere of influence and/or being limitedly dependent on other parties (e.g., the municipal college and council, rules at national level). | Save “stuff” for the results display. |
| 8.3 Available hours | Having hours available to execute HWA tasks. | Planning which instructor/physiotherapist will follow/provide [project name]. |
| 8.4 Compatible tasks | Having compatible tasks to execute HWA tasks. |  |
| 9.1 Spur other professionals into action | Having some professionals who spur other professionals into action to work on the HWA, for example by coordinating activities, taking the lead in an action, or chairing a working group. | Invite these people during a next meeting to go more in-depth: How? What can you actually do yourself? |
| 10.1 Look for entry point | Looking for an entry point into the target group, for example via key persons (e.g., care professionals) or locations (e.g., community centers, sports associations, or schools). | Making contact with part of target group to set up a cooking and walking club for non-western ladies together with the dietician. |
| 10.2 Existing contact points with care professionals | Use existing contact points with care professionals to talk with citizens about lifestyle or HWA activities. | Discuss lifestyle with clients during treatment sessions. |
| 10.3 Referrals from care professionals | Care professionals refer citizens with overweight to other professionals. | Arrange feedback from meeting group: did they participate [in the project name*]?* Experiences? Anything needed for security? This in coordination with the municipality. |
| 10.4 Determine target groups’ needs | Determine citizens’ needs in order to adjust the HWA accordingly. | Full of energy, we continue with the actions that are planned in [name municipality] to get even more in touch with the citizens about where their needs lie. Discuss this with a team of neighborhood sports coaches and colleagues in social cultural work. |
| 11.1 Strengthen support base | Strengthening support among citizens regarding overweight and the HWA, so citizens perceive overweight and/or the HWA as important or support it themselves (e.g., citizens want to participate in activities in their own municipality). | Looking for reasons why people/athletes continue to exercise with us, or stop/do not attend. |
| 11.2 Low entry threshold | Give citizens a push to facilitate participation in an activity and/or to engage in a healthy lifestyle by having low entry thresholds (e.g., smaller participation fee may lower citizens’ entry threshold). | [Project name] or at another place to bring story to target group |
| 11.3 Customization | Customizing the HWA to facilitate participation by tailoring HWA elements such as activities to a citizen’s personal situation and preferences. | How to better support migrants (0–4 years)? |

^a^ LPTs in the original article are described in terms of how they were experienced in 2021 (Ter Bogt et al., 2023). In the current table, these LPTs are described in terms of the needed leverage direction. Therefore, some LPTs are rephrased (e.g., slow process 🡪 faster process).

| **ASM level (Nobles et al., 2021)** | **Definition (Nobles et al., 2021)** | **Operationalization** | **Example action** |
| --- | --- | --- | --- |
| **Events** | Action is focused mainly on *observable behaviors and outcomes of stakeholders within the system (e.g., symptoms of the system).* | Includes mainly building up, adapting, or performing activities, interventions, or facilities for the target group or among professionals. | Organize another prevention cafe. |
| **Structures** | Action is focused mainly on *the organization of the system that causes these events to occur (e.g., physical structures, patterns, relations, information streams).* | Includes mainly the organization to make [events] happen, such as organization structures, patterns, relationships, information flows, or collaborations. | Contact the organizer of the idea presented and ask if she can help with a similar project in [name municipality]. |
| **Goals** | Action is focused mainly on the *ambitions toward which the systems work*. | Includes mainly the (small or big) formulated goals of the individual or organization within the system or the system as a whole and toward which they work. For example, actions can adapt existing goals or cover a new goal. | Still focus on [project name], despite the fact that partners now have less energy for this. Look for other parties. |
| **Beliefs** | Action is focused mainly on *deeply held norms, attitudes, and values about elements of the system.* | Includes mainly norms, attitudes, and values that underly the system’s goals. Therefore, it covers mainly why something is done. It also includes convincing others. | Consultation with municipalities on the embedding of policy and finance for the purpose of a structural approach: trying to sharpen the goals, resources, and profit for policymakers so that expenditures now become profit in the future. |
